# Case report: acquired Gerbode defect and ventricular septal defect in a patient with native valve infective endocarditis

**DOI:** 10.1093/ehjcr/ytag172

**Published:** 2026-03-18

**Authors:** Michael Gomes, Jeni Jones, Jonathan Teoh, Dale Currigan, Graham Jenkins

**Affiliations:** Joondalup Health Campus, Shenton Avenue, Joondalup, WA 6027, Australia; Joondalup Health Campus, Shenton Avenue, Joondalup, WA 6027, Australia; Joondalup Health Campus, Shenton Avenue, Joondalup, WA 6027, Australia; Sir Charles Gairdner Hospital, Hospital Avenue, Nedlands, WA 6009, Australia; Joondalup Health Campus, Shenton Avenue, Joondalup, WA 6027, Australia

## Abstract

**Background:**

Infective endocarditis (IE) is a life-threatening condition with significant morbidity and mortality. Complications, such as abscess formation, fistulae, and intracardiac shunts, including Gerbode defects, are rare but critical to recognize.

**Case summary:**

A 67-year-old male with severe aortic stenosis and a bicuspid aortic valve presented with palpitations, fevers, and vomiting. After progression of his symptoms and the development of cardiogenic shock, investigations revealed *Streptococcus mitis* IE of the aortic and tricuspid valves, complicated by aortic root abscess, complete heart block, and a Gerbode defect with a ventricular septal defect. Diagnosis was confirmed with transoesophageal echocardiography, and urgent surgical intervention included valve replacement, tricuspid valve repair, and closure of the defects. The patient was discharged with a 6-week course of intravenous antibiotics and multidisciplinary follow-up.

**Discussion:**

This case highlights the diagnostic and therapeutic challenges of IE, underscoring the importance of imaging and early surgical intervention. Gerbode defects, though rare, are increasingly recognized as severe complications of IE, necessitating prompt management.

Learning points

- A Gerbode defect is a rare left ventricle–right atrium communication, often due to fistulizing infective endocarditis, and must be promptly recognized to prevent acute haemodynamic compromise.
- Patients with bicuspid aortic valve and severe aortic stenosis are at moderate risk of infective endocarditis; early blood cultures are essential to avoid a delayed diagnosis.

## Introduction

Infective endocarditis (IE) is a serious condition associated with significant morbidity and mortality, particularly among patients with pre-existing valvular heart disease. Diagnosis of IE is not easy and this case highlights the diagnostic challenges and rapid progression of aortic valve IE in a patient with severe aortic stenosis and a bicuspid aortic valve, culminating in atrioventricular block, abscess formation, and an acquired Gerbode fistula. This is a rare but critical complication emphasizing the importance of early recognition and prompt investigation.

A Gerbode defect refers to an abnormal communication between the left ventricle (LV) and right atrium (RA). Although such LV–RA shunts were recognized pathologically as early as the nineteenth century, the lesion was first clinically characterized and surgically repaired by Gerbode *et al.*,^1^ in 1958, described its distinctive anatomy and haemodynamic profile. Whilst classically congenital, Gerbode defects may also be acquired, most often secondary to infective endocarditis, cardiac surgery, or trauma.^2^

## Summary figure

**Table:** 

Day 0—presented to the emergency department with subjective fevers and nausea.
Day 0—discharged home after being diagnosed with a lower respiratory tract infection, with an elevated white cell count (11.8 × 10^9^/L) and C-reactive protein (108 mg/L), a normal chest X-ray, and a negative respiratory viral PCR panel.
Day 14—developed palpitations, fatigue, and vomiting. Represented to the emergency department feeling much worse. His C-reactive protein had increased to 216 mg/L, and WCC 23. Commenced empirical antibiotics.
Day 15—patient deteriorated in coronary care unit with cardiogenic shock due to complete heart block. An isoprenaline infusion was commenced. An urgent TTE demonstrated aortic and tricuspid vegetations.
Day 15—a TOE indicated the presence of an aortic root abscess with rupture, causing a Gerbode defect and VSD.
Day 16—surgical intervention with aortic and tricuspid vegetectomy, aortic valve replacement, tricuspid valve repair and a bovine pericardial patch was used for patch closure of the VSD and Gerbode defect.
Day 17—permanent pacemaker implanted.
Day 21—discharged from hospital with PICC line for 6 weeks of intravenous antibiotics.
Day 56—completed 6 weeks of intravenous antibiotics.

## Case presentation

A 67-year-old male with a bicuspid aortic valve and severe aortic stenosis presented to the emergency department with palpitations and subjective fevers. The patient was under regular cardiology follow-up, with planned 6-monthly reviews and consideration for valve intervention at the onset of symptoms.

The patient presented with subjective fevers, exertional dyspnoea, an elevated white cell count (WCC) of 11.8 × 10^9/L, and an elevated C-reactive protein of 108 mg/L. Auscultation revealed right lower zone crepitations. The respiratory viral PCR, urine culture, and chest X-ray were unremarkable. No blood cultures were obtained at this stage. With no objective fevers recorded in the emergency department and no further nausea or vomiting, he was discharged with oral antibiotics for a lower respiratory tract infection.

Two weeks later, the patient presented to the emergency department. He reported significant fatigue after mowing his lawn earlier that day, followed by nausea and two episodes of vomiting. There had been continuation of his subjective fevers. The patient was diaphoretic and exhibited intermittent Wenckebach and 2:1 atrioventricular block.

Within 2 h, the patient showed signs of clinical deterioration, including worsening conduction abnormalities with intermittent complete heart block and a ventricular escape rhythm of 44 beats per minute. Laboratory investigations revealed a WCC of 23 × 10^9/L and a C-reactive protein of 216 mg/L. Physical examination identified a loud ejection systolic murmur radiating to the carotid arteries, with a clear chest on auscultation. Notably, the patient had poor oral health, raising suspicion for a dental source of infection.

Given the new conduction abnormalities and clinical presentation, a provisional diagnosis of infective endocarditis was made. The infectious diseases team was consulted, and the patient was commenced on empirical antibiotics (ceftriaxone and vancomycin).

Within 24 h of admission, the patient’s condition worsened, with declining blood pressure, reduced urine output, and a further drop in his ventricular escape rate to the 30 s. An arterial line was inserted, and an isoprenaline infusion was initiated for haemodynamic support.

**Figure 1:**
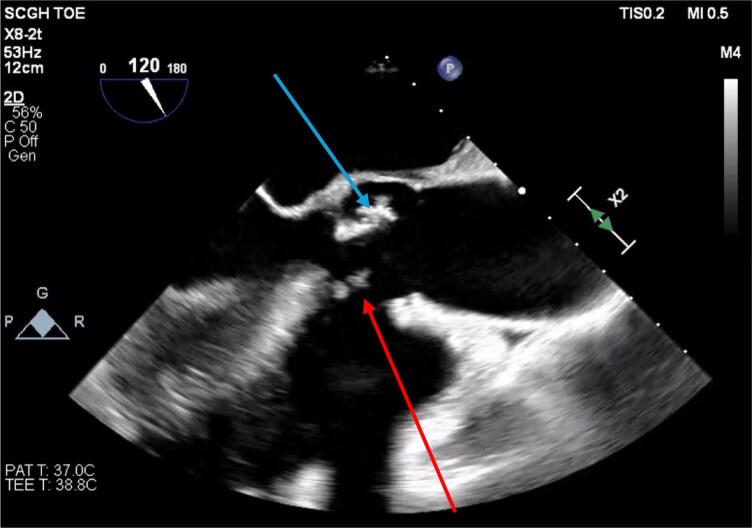
Intraoperative transoesophageal echocardiography image in the mid-oesophageal long-axis view (120°) demonstrating the aortic root and ventricular septal defect. The blue arrow indicates vegetation on the aortic valve, whilst the red arrow identifies the ventricular septal defect.

**Figure 2:**
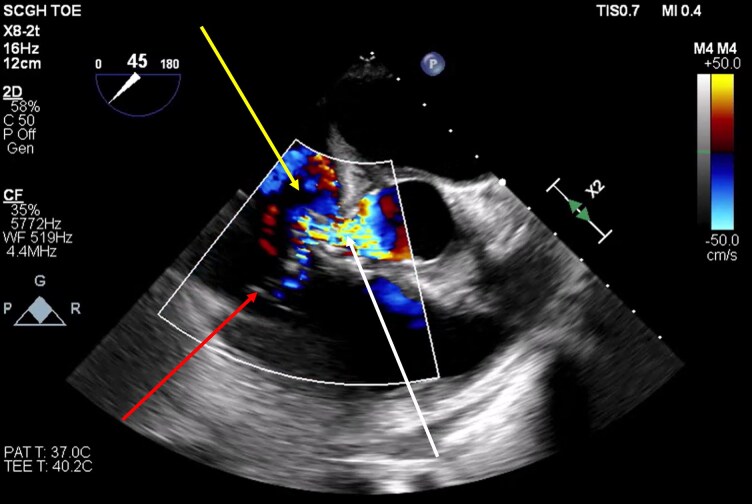
Intraoperative transoesophageal echocardiogram. Mid-oesophageal aortic valve short-axis view (45°): shows the jet’s origin near the non-coronary cusp (white arrow) and its direction into the right atrium (yellow arrow), confirming the presence of a Gerbode defect. The tricuspid valve is indicated by a red arrow.

**Figure 3:**
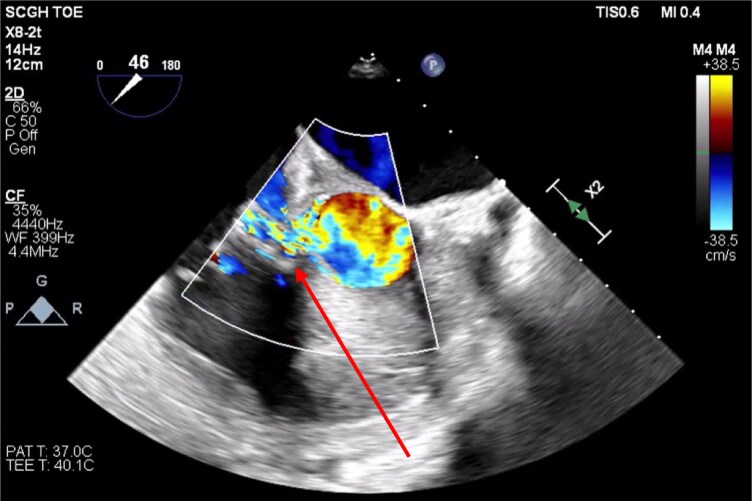
Intraoperative transoesophageal echocardiogram image short axis showing colour Doppler of flow across the Gerbode defect into the right atrium, superior to the tricuspid annulus (red arrow).

**Figure 4:**
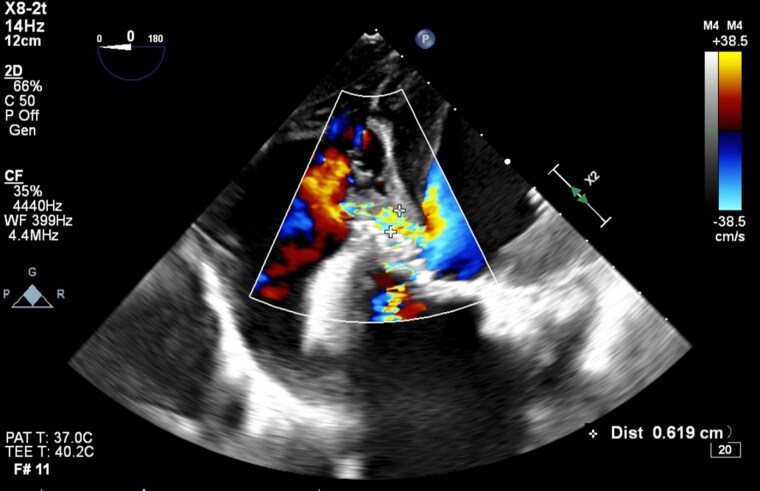
Intraoperative transoesophageal echocardiogram image showing flow across ventricular septal defect with a diameter measured at 0.619 cm. Jet location indicated by white crosses.

A transthoracic echocardiogram (TTE) demonstrated multiple independently mobile echodensities on the aortic and tricuspid valves, consistent with vegetations. The TTE also revealed mild to moderate aortic regurgitation and a severely stenotic aortic valve with an area of 1 cm^2^ and a mean transvalvular gradient of 42 mmHg. These findings were confirmed on a transoesophageal echocardiogram (TOE). Additionally, there was evidence of an aortic root abscess extending into the membranous septum, rupturing and causing a communication between the LV and the RA, a finding consistent with a Gerbode defect (*Figures 1* and *2*), and a 1.5 × 1.8 cm perimembranous ventricular septal defect (VSD) (*Figures 3* and *4*).

**Figure 5:**
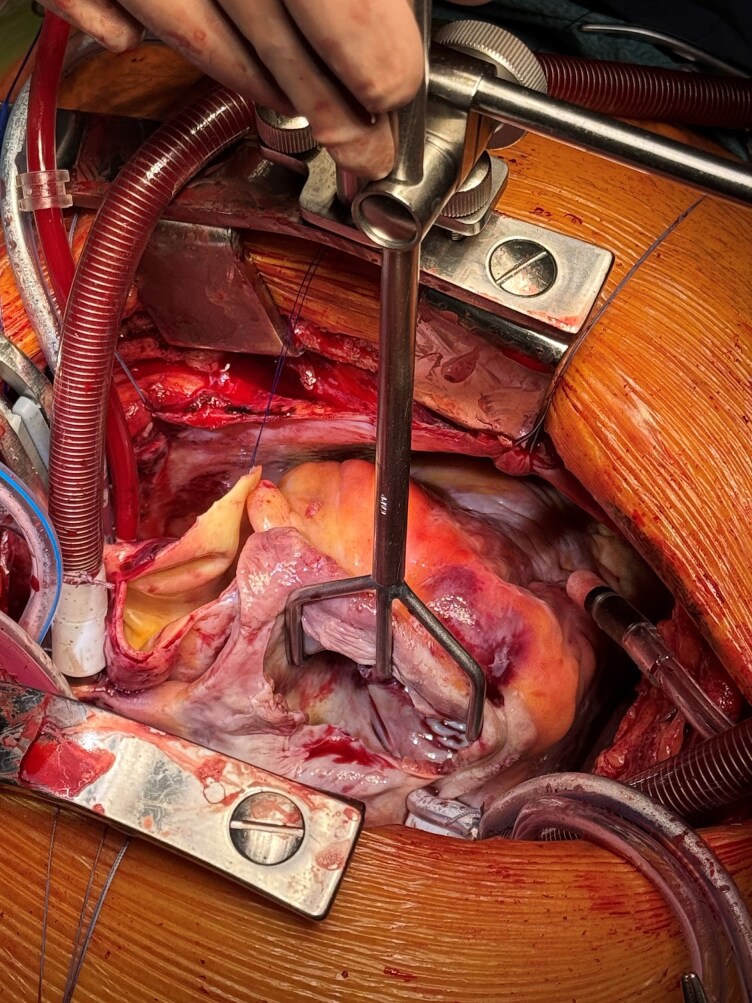
Intraoperative photograph demonstrating the patient on cardiopulmonary bypass with the right ventricle opened. The operative field shows the exposure of the interventricular septum and adjacent structures following establishment of bypass and cardioplegic arrest.

Urgent cardiothoracic surgical consultation was sought, and the patient was transferred to a specialized cardiothoracic centre. A temporary pacing wire had been considered prior to transfer, but this was avoided with the patient remaining haemodynamically stable on an isoprenaline infusion. Blood cultures taken at presentation grew *Streptococcus mitis* in all three sets. Given the presence of aortic root abscess, severe valvular dysfunction, and the development of a Gerbode defect, the decision was made to proceed with emergent surgical intervention the following morning. The patient underwent bioprosthetic aortic valve replacement, tricuspid valve vegetectomy, and repair, and a bovine pericardial patch was used for patch closure of his VSD and Gerbode defect (*Figure 5*). A dual-chamber transvenous permanent pacemaker was inserted the following day.

Our patient was then discharged with a 6-week course of intravenous antibiotics, with follow-up arranged with his cardiologist, his cardiothoracic surgeon, and infectious diseases physician. He subsequently completed 6 weeks of intravenous antibiotics without further complications.

## Discussion

Infective endocarditis is an uncommon disease with high morbidity and mortality, with a 30-day mortality rate of up to 30%.^3^ Complications of untreated infective endocarditis can be fatal. Clinical presentation is variable, and diagnosis incorporates high clinical suspicion, the modified DUKE criteria, and imaging findings.^4–6^ Rapid diagnosis, initiation of empirical antibiotics, and recognition of complications are essential to improve patient outcomes.^5^ Our case highlights the importance of considering infective endocarditis, and its potential mechanical complications, even in patients with native valves.

Periannular extension of IE can occur in 10%–40% of cases and is most commonly associated with aortic valve.^4,5^ Such complications correspond to higher mortality rate.^4,5^ Native aortic valve complications, such as abscess and fistulization, often arise at the weakest part of the annulus, located close to the septum and atrioventricular node. Thus, new conduction abnormalities are a strong predictor of complication.^3,6^ Electrocardiogram (ECG) is a widely available and inexpensive tool that has an important role, as seen in this case.

A key teaching point is the missed opportunity for early investigation. This patient had a known bicuspid aortic valve with severe stenosis, which places him at moderate risk of IE according to the European Society of Cardiology (ESC) guidelines.^5^ At his initial emergency department presentation with fever and elevated inflammatory markers, blood cultures should have been obtained promptly. Failure to do so delayed the recognition of IE and subsequent definitive management.

The 2023 ESC guidelines recommend TTE as the initial imaging modality.^7^ Whilst TTE is a rapid and non-invasive diagnostic tool, the detection of perivalvular complications is low.^5^ Transoesophageal echocardiogram provides a significantly increased sensitivity (76%–100%) and specificity (95%) for perivalvular extension of IE and is recommended in the ESC guidelines unless there is solely right-sided disease with unambiguous findings.^7^ This is a result of direct transducer locality to the aortic root and basal septum, where abscess and fistulae commonly occur. In our case, TOE demonstrated a fistula, giving rise to a communication between the aortic root and LV, a Gerbode defect. Transoesophageal echocardiography is the imaging modality of choice. Characteristic features include a high-velocity left-to-right jet originating from the upper membranous septum and directed towards the right atrium, best visualized on colour Doppler. The shunt is typically present throughout systole and persisting into early diastole, reflecting the continuous pressure gradient from the LV to the RA across the cardiac cycle. Intracardiac shunts as a result of fistulae can be catastrophic in IE and require urgent surgical intervention.^4,5^

Gerbode defects account for 0.08% of intracardiac shunts and <1% of congenital heart abnormalities.^8^ Whilst most Gerbode defects are congenital in origin, cases of acquired defects are increasing.^8^ Acquired Gerbode defects are often iatrogenic, arising from previous cardiac surgery or invasive cardiac procedures.^8^ Most non-iatrogenic cases of acquired Gerbode defect are secondary to IE, with findings increasingly recognized in the last 10 years.^8^

Urgent cardiac surgery for complex IE is recommended across international guideline bodies.^4,7^ Valve replacement or repair and closure of fistula tracts are the definitive surgical options.^4^ Subsequent mortality remains high,^9^ and patients will require long-term follow-up and education.^4^

Whilst surgical repair remains the standard of care for most acquired Gerbode defects, particularly in the context of active infective endocarditis, alternative treatment options have been increasingly reported. Percutaneous transcatheter closure using occluder devices—such as Amplatzer membranous VSD or duct occluders—has emerged as a viable option in selected stable patients without active infection or extensive tissue destruction. This minimally invasive approach may be considered in small residual or iatrogenic shunts, or in patients deemed high risk for surgical reoperation. Successful closure has been achieved under TOE or fluoroscopic guidance with low procedural morbidity and good mid-term outcomes.^10–12^ However, in the setting of ongoing infection, medical therapy alone is inadequate, and surgical intervention remains essential to eradicate infection, debride necrotic tissue, and restore structural integrity. The choice of therapy should therefore be individualized, guided by shunt size, haemodynamic significance, presence of infection, and patient comorbidity.

In conclusion, IE and its complications should be considered even in those with native valves. Significant perivalvular progression can result in abscess formation, fistula, and intracardiac shunting and complete heart block.^1,2^ Prompt recognition and management are essential to successful patient outcomes.^3^ Ongoing research for IE may allow improved knowledge, therapy, and prophylaxis to help reduce patient morbidity and mortality.^3^

## Lead author biography


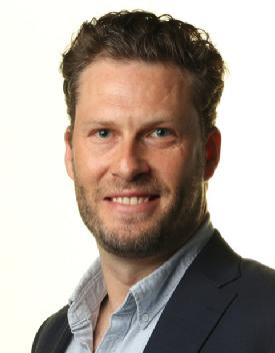


Michael Gomes, MBBS, is a medical registrar and chief registrar at Royal Darwin Hospital, Northern Territory, Australia. He completed his medical degree at the University of East Anglia, UK, and has worked across tertiary and regional centres in both the UK and Australia. Michael has a strong academic interest in cardiology, with experience in complex cardiac imaging and structural heart disease. He is also completing a Master of Medicine (Internal Medicine) at the University of Sydney, with a focus on clinical research, and is pursuing advanced training in cardiology.

## Data Availability

The data underlying this article are available in the article and in its online supplementary material.

## Notes

**Consent:** Written informed consent was obtained from the patient for publication of this case report and accompanying images, in compliance with the COPE guidelines.
